# Funny Excuses

**Published:** 1898-10

**Authors:** 


### Funny Excuses.

A school teacher with an eye for the humorous sends in the following collection of funny excuses that have fallen in her way while teaching:

Miss Brown,—You must stop teach my Mabel fisical torture, she needs yet readin' and figors mit sums more as that, if I want her to do jumping I kin make her jump. Mrs. Cana vowski.

Miss Blank,—Please excuse my Earl for bein' absent he is yet sick with dipterry and der doctors don't think he will discover to oblige his loving aunt Mrs.——. I am his mother's sister from her first husband.

Miss,—Frank could not come these three wks, cause he had the information of the vowels. Mrs. Smith.—*Ex.*

